# Integrating Community Engagement in Zero Leprosy Efforts: A Pathway to Sustainable Early Detection, Control and Elimination

**DOI:** 10.3390/tropicalmed9120296

**Published:** 2024-12-03

**Authors:** Anil Fastenau, Matthew Willis, Constanze Vettel, Sophie C. W. Stuetzle, Srilekha Penna, Priyanka Chahal, Fabian Schlumberger, Mowmita Basak Mow, Ngozi Ekeke, Joseph Ngozi Chukwu, Patricia D. Deps

**Affiliations:** 1 German Leprosy and Tuberculosis Relief Association (GLRA/DAHW), 97080 Wurzburg, Germany; anil.fastenau@dahw.de (A.F.); constanze.vettel@dahw.de (C.V.); ngozi.ekeke@redaid-nigeria.org (N.E.); joseph.chukwu@dahw.org (J.N.C.); 2 Marie Adelaide Leprosy Center (MALC), Karachi 74400, Pakistan; sophie.stuetzle@web.de (S.C.W.S.); fabian@malc.org.pk (F.S.); 3 German Leprosy and Tuberculosis Relief Association India (GLRA India), New Delhi 110092, India; srilekha.penna@glraindia.in; 4 Department of Global Health, Institute of Public Health and Nursing Research, University of Bremen, 28359 Bremen, Germany; 5 Heidelberg Institute of Global Health (HIGH), Medical School, Heidelberg University, 69120 Heidelberg, Germany; 6 School of Medicine, Dentistry & Biomedical Sciences, Queen’s University Belfast, Belfast BT7 1NN, UK; 7 Department of Global Health and Social Medicine, Harvard Medical School, Boston, MA 02115, USA; priyanka_chahal@hms.harvard.edu; 8 Lepra, Colchester CO1 1TB, UK; mowmitab@lepra.org.uk; 9 RedAid Nigeria, Enugu 400001, Nigeria; 10 Department of Social Medicine, Federal University of Espírito Santo, Vitória 29075-910, ES, Brazil; patricia.deps@ufes.br

**Keywords:** community engagement, leprosy, Zero Leprosy, leprosy elimination, community-based approaches

## Abstract

Community engagement has emerged as a critical component in the effective control and elimination of neglected tropical diseases (NTDs), particularly in regions with persistent stigma and limited healthcare access. Drawing on case studies from Brazil, India, and Nigeria, this opinion piece explores how community-driven initiatives have successfully improved leprosy awareness, reduced stigma, and fostered early case detection and treatment adherence. The importance of culturally sensitive, inclusive approaches in health education and stigma reduction campaigns is highlighted, emphasizing the potential for community engagement to enhance national leprosy programs and contribute to the World Health Organization’s Zero Leprosy Strategy. By examining these examples, this article illustrates how integrating community participation into leprosy control and elimination programs can drive sustainable outcomes for achieving Zero Leprosy, even in resource-limited settings.

## 1. Introduction

Leprosy, also known as Hansen’s disease, is an infectious neglected tropical disease (NTD) caused by *Mycobacterium leprae* [1]. Despite significant advancements in diagnosis and treatment, it continues to persist in several endemic regions, remaining stigmatized and frequently misunderstood [1,2]. The continued high prevalence of leprosy and leprosy-related disabilities in low-resource settings underscores the need for innovative strategies to enhance active case finding and early detection to stop the transmission [3,4]. Research indicates that community engagement has increasingly shown promising results in strengthening leprosy control and elimination programs, especially in areas where social stigma and a lack of health infrastructure hinder early diagnosis, treatment, and interruption of transmission [5]. Community engagement in leprosy programs involves empowering communities through culturally sensitive education, collaboration with local influencers, training community health workers, and engaging individuals affected by leprosy as champions to promote awareness, reduce stigma, and improve early diagnosis and treatment outcomes. This opinion manuscript discusses the potential of community engagement as a cornerstone of sustainable leprosy control and elimination efforts. By drawing on case studies from leprosy high-endemic countries Brazil, India, and Nigeria, we aim to illustrate how empowering communities not only improves early detection rates but also fosters an environment where leprosy can be openly discussed without fear or prejudice. The focus is on exploring culturally inclusive strategies, health education, and local partnerships that can be integral to leprosy programs. By integrating these community-based approaches into leprosy control efforts, we argue that endemic countries can make significant strides toward the goals of the World Health Organization’s Zero Leprosy Strategy.

## 2. The Rationale for Community Engagement in Leprosy Control and Elimination

In leprosy-endemic countries, the success of leprosy control and elimination programs is often hindered by a lack of awareness, cultural misconceptions, and deep-rooted stigma [6]. Unlike other neglected tropical diseases (NTDs), leprosy carries a unique burden of social stigma and discrimination, which, in turn, often results in delayed diagnosis and treatment, eventually causing severe disabilities [7]. Community engagement directly addresses these barriers by fostering a supportive environment where leprosy is understood as a treatable disease rather than a source of fear and social exclusion [8]. Community-driven initiatives can also provide cost-effective solutions in low-resource settings [9]. By training local health workers, mobilizing community leaders, and engaging individuals affected by leprosy in advocacy, communities take ownership of leprosy control and elimination efforts, becoming active participants in the process [10]. This approach empowers communities to improve healthcare access and drive sustainable change [10]. Furthermore, this model can improve healthcare access and help reduce the reliance on external resources, making it a sustainable approach that is essential for long-term success in achieving Zero Leprosy [11].

## 3. Case Studies of Successful Community Engagement in Leprosy Control

### 3.1. Brazil

In Brazil, community health agents play an essential role in leprosy control programs. Trained in identifying early signs and symptoms of Hansen’s disease, these agents work within their communities to raise awareness and refer suspected cases to health facilities [12]. Research from Brazil indicates that actively involving community agents can significantly boost early diagnosis rates, enabling timely treatment and reducing transmission of Hansen’s disease [13]. This model of embedding community health workers into leprosy programs underscores the value of localizing healthcare delivery [11].

### 3.2. India

India’s National Leprosy Eradication Programme (NLEP) has also integrated community engagement strategies in several states. In regions like Bihar, community members are trained to identify leprosy symptoms, conduct health education sessions, and reduce stigma through personal testimonies [14]. The inclusion of people affected by leprosy as champions of the disease has transformed social attitudes, with local leaders actively participating in leprosy awareness campaigns [15]. These efforts have led to increased reporting of suspected leprosy cases and have helped bridge the gap between communities and healthcare providers in India [15].

### 3.3. Nigeria

Nigeria’s innovative approach to community engagement involved partnering with local religious leaders to address leprosy-related stigma [16]. In many communities, religious leaders hold significant influence and can play a crucial role in reshaping attitudes toward leprosy [17]. By involving these influential leaders in educational workshops, the leprosy control programs can successfully reduce stigma and increase community acceptance of individuals affected by leprosy [16]. As a result, more individuals seek early treatment without fear of discrimination, improving the overall success of leprosy control and elimination measures.

## 4. Key Components of Successful Community Engagement in Leprosy Programs

Drawing from these case studies, several key components emerge as fundamental to successful community engagement in leprosy control:

### 4.1. Education and Awareness

Comprehensive, culturally sensitive health education can demystify leprosy, dispel myths, and encourage early leprosy treatment. Community-led sessions allow individuals to ask questions, understand symptoms, learn about the benefits of early diagnosis, and help reduce stigma surrounding the disease [18]. Additionally, integrating local and traditional practices into education efforts can enhance their effectiveness and adherence. By acknowledging and leveraging these practices, health initiatives can resonate more deeply with targeted populations, fostering trust and improving engagement.

### 4.2. Collaboration with Influential Leaders

Working with local influencers—such as religious leaders or local government officials—ensures wider acceptance and reach of leprosy initiatives. These partnerships validate leprosy elimination programs in the eyes of the community and enhance trust [19]. Participants in these communities reported significant reductions in personal restrictions, highlighting the positive impact of engaging respected community figures [19].

### 4.3. Training of Community Health Workers (CHWs)

CHWs are often the first point of contact for individuals in remote areas. By equipping them with skills to identify leprosy symptoms and conduct awareness sessions, CHWs can significantly improve early case detection and follow-up care in communities with limited healthcare access [20].

## 5. Challenges and Considerations in Community Engagement

While community engagement offers immense potential, implementing these programs comes with challenges. Funding constraints, community resistance, and logistical barriers can hinder the reach and efficacy of community engagement initiatives. There is also a need for continuous training, capacity strengthening, and support for community health workers and local champions to ensure that knowledge is retained and updated. Furthermore, community engagement must be tailored to local cultural contexts. A strategy that works in one country or region may not be directly transferable to another. Programs must adapt to the specific needs, beliefs, and social dynamics of each community, requiring ongoing collaboration and flexibility. Additionally, the knowledge and influence of elderly community members, along with traditional practices, can be invaluable in shaping culturally appropriate and effective engagement strategies.

## 6. Recommendations for Future Leprosy Control and Elimination Programs

To maximize the impact of community engagement, leprosy control and elimination programs should incorporate the following recommendations:

**1. Expand Training for CHWs:** Increasing the number and skills of community health workers will enhance outreach and improve early detection rates, especially in hard-to-reach remote areas.

**2. Promote Inclusion of Persons affected by Leprosy:** Encouraging people affected by leprosy who have successfully completed treatment to advocate for leprosy awareness can humanize the disease, reduce social stigma, and enhance early diagnosis.

**3. Strengthen Monitoring and Evaluation:** Implementing robust monitoring systems will help track the impact of community engagement efforts and identify areas for improvement. These data can be invaluable for scaling successful community-led interventions.

**4. Secure Sustainable Funding Sources:** Community engagement requires long-term commitment. By securing funding from diverse sources—including government, NGOs, and private sector partnerships—leprosy control and elimination programs can ensure sustainability and reduce dependence on temporary grants.

**5. Support Research on Community Engagement:** Investing in research focused on community engagement and community-led projects will provide valuable insights into best practices, enhance understanding of effective strategies, and support evidence-based decision-making to improve leprosy control and elimination programs.

## 7. Conclusions

Community engagement stands as a vital element in the path towards Zero Leprosy. By integrating community participation into leprosy control and elimination programs, countries can foster early detection, reduce stigma, and empower local populations to take an active role in disease control. The success of community engagement in Brazil, India, and Nigeria demonstrates that community-driven approaches can lead to sustainable outcomes in leprosy control. As the World Health Organization’s Zero Leprosy Strategy progresses, the active involvement of communities will be indispensable in ensuring that no one is left behind in the global efforts to eliminate leprosy.

## Data Availability

Not applicable.
